# Comparing Acute Effects of Caffeine Delivery Forms on Cross-Training Performance: A Randomized Placebo-Controlled Crossover Trial

**DOI:** 10.3390/nu18040657

**Published:** 2026-02-17

**Authors:** Salvador Vargas-Molina, Diego A. Bonilla, Manuel García-Sillero, Sergio Iglesias-Placed, Mora Murri, Fernando Martín-Rivera, Javier Benítez-Porres

**Affiliations:** 1 Department of Human Physiology, Physical Education and Sport, Faculty of Medicine, University of Malaga, 29010 Malaga, Spain; salvadorvargasmolina@gmail.com (S.V.-M.); manugarciasillero@gmail.com (M.G.-S.); 2 Research Group in Sports Nutrition (DBSS-Nut), Dynamical Business & Science Society—DBSS International SAS, Bogotá 110311, Colombia; dabonilla@dbss.pro; 3 Hologenomiks Research Group, Department of Genetics, Physical Anthropology and Animal Physiology, University of the Basque Country (UPV/EHU), 48940 Leioa, Spain; 4 EADE, Trinity Saint David, C/Miguel Sel Gómez de la Cruz, 2, 29018 Malaga, Spain; sergioiglesias@eade.es; 5 Instituto de Investigación Biomédica de Málaga y Plataforma en Nanomedicina-IBIMA Plataforma BIONAND, 29590 Malaga, Spain; moramurri@gmail.com; 6 Cardiology and Cardiovascular Surgery Clinical Management Unit (UGC), Virgen de la Victoria University Hospital, Campus de Teatinos s/n, 29010 Malaga, Spain; 7 Endocrinology and Nutrition UGC, Victoria Virgen University Hospital, 29010 Malaga, Spain; 8 CIBER Physiopathology of Obesity and Nutrition (CIBEROBN), Carlos III Health Institute, 28029 Madrid, Spain; 9 Research Unit in Sports and Health, University of Valencia, Gascó Oliag, 3, 346010 Valencia, Spain; fernando.martin-rivera@uv.es; 10 Internal Medicine Clinical Management Unit (UGC), Hospital Regional Universitario de Malaga, 29010 Malaga, Spain

**Keywords:** high-intensity functional training, dietary supplements, chewing gum, mouth rinse, ergogenic aids

## Abstract

**Background/Objectives**: The aim of this study was to compare the different forms of caffeine (CAF) administration in CrossFit^®^ participants. The countermovement jump (CMJ), the rate of perceived exertion (RPE), the total number of repetitions, and the maximum (HR_max_) and mean heart rate (HR_mean_) were evaluated. **Methods**: Fourteen males with more than six months of continuous CrossFit^®^ training (30.9 [5.62] years, 179 [1.33] cm, 78 [5.75] kg, 24.3 [1.33] kg·m^−2^) participated in this randomized, placebo-controlled, crossover study. Participants were randomized in a repeated measures design using caffeine capsule (CC), caffeine chewing gum (CCG), and caffeine mouth rinse (CMR) protocols, along with a placebo group (PG). Participants were unaware of whether any of the delivery methods contained caffeine. A 7-day washout period before each crossover was used. To ensure ecological validity, we replicated the real-world practice of CAF ingestion 30 min prior to training, mirroring typical athlete pre-workout routines. The participants of CrossFit^®^ performed the ‘Cindy’ protocol, and the CMJ as a primary outcome was measured pre- and post-intervention, while the RPE, HR, and the number of repetitions were tracked at the end of the workout for comparisons. **Results**: No significant differences were found between CAF forms in internal load measures (RPE, HR_max_, HR_mean_) or the number of repetitions. While no changes were observed with other CAF forms, CMR significantly improved the CMJ performance compared to the baseline (Δ: +3.5; Cohen’s d_unb_: 0.51], which exceeded the estimated SWC by approximately three-fold. However, the inferential analysis revealed no significant main effects of the caffeine administration method on any measured outcomes. **Conclusions**: While CAF delivery forms did not improve internal load measures or performance parameters, such as the RPE, HR, or the total number of repetitions, the caffeine mouth rinse (CMR) showed a potentially meaningful improvement in CMJ performance after CrossFit^®^ training in participants with a certain level of experience.

## 1. Introduction

Caffeine (CAF), a methylxanthine derivative, acts as a central nervous system stimulant with rapid gastrointestinal absorption [1]. Over 95% of CAF undergoes hepatic metabolism via the cytochrome P450 1A2 (CYP1A2) enzyme [2]. Like other methylxanthines (theophylline and theobromine), CAF exerts psychostimulant effects, potentially through enhanced norepinephrine secretion [3], thereby promoting alertness, reducing fatigue, and serving as an ergogenic aid in high-intensity training modalities like CrossTraining (e.g., CrossFit^®^). Research demonstrates CAF’s performance-enhancing effects across endurance sports, including cycling [4,5], swimming [6], or athletics [7,8], with documented improvements in oxygen uptake (VO_2max_), respiratory exchange ratios, and ratings of perceived exertion (RPE) [9]. While commonly consumed via coffee, tea, yerba mate, and chocolate, an ongoing scientific debate persists regarding optimal administration forms and the bioavailability required to elicit maximal ergogenic benefits.

On the one hand, CAF administration via chewing gum (CCG) demonstrates dual absorption through both intestinal and oral mucosal pathways [10], with a potential higher bioavailability compared to traditional capsules [11]. While CCG has shown performance benefits in cyclists [12,13] and football players [14], data remain limited for strength-trained populations. Similarly, caffeine mouth rinsing (CMR) has yielded positive effects in endurance cycling [15] and sprint power outputs [16], though CCG applications in resistance-trained participants showed no ergogenic benefit for the total load lifted [17]. Notably, research has identified a dose–response placebo effect when participants believed they were consuming caffeine [18], prompting our inclusion of a placebo control group.

Existing CrossFit^®^ studies have exclusively used capsules administered 50–70 min pre-test (3–9 mg/kg body mass) [19,20,21,22], showing no benefits for the internal load (RPE, HR) or performance metrics like the number of repetitions. The aim of our study is to compare CAF’s ergogenic effects across administration methods (capsules, chewing gum, and mouthwash) in CrossFitters. We hypothesized that the caffeine administration method would differentially influence performance and internal load responses during a standardized CrossFit^®^ workout, with faster delivery methods (chewing gum and mouth rinse) showing greater effects compared with capsules and the placebo.

## 2. Materials and Methods

**Figure 1:**
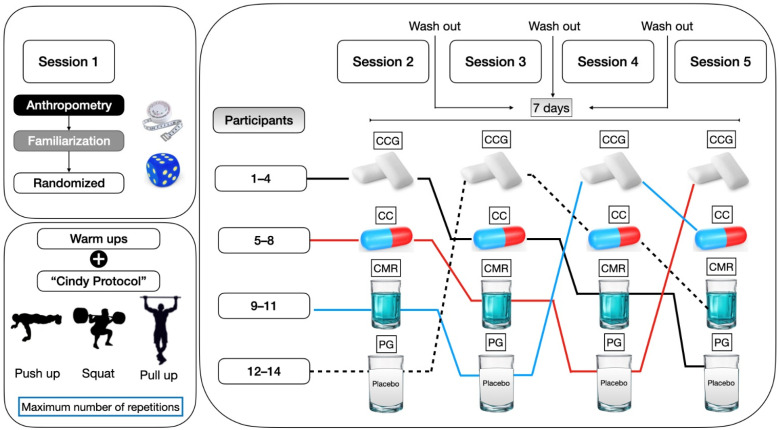
Schematic representation of the study design. CCG: caffeine chewing gum; CC: caffeine capsule; CMR caffeine mouth rinse; and PG: placebo group.

This was a randomized, blind, crossover study of participants. As inclusion criteria, participants had to train at least three times per week with a minimum duration of 60 min per session. Subjects were considered healthy, with no medical diagnosis of any type of illness. All subjects maintained stable dietary and training habits throughout the experimental period; were not following extreme diets or severely energy-restricted programs; and were not in a period of injury, rehabilitation, or deload. Additionally, they did not consume any supplements containing stimulants, including caffeine. CrossFit^®^ participants with more than six months of continuous training were randomly assigned to the following protocols: CAF capsules (CCs), CAF chewing gum (CCG), CAF mouth rinse (CMR), or placebo (PG). At no point during this study were participants informed whether a caffeine-free protocol was available. Therefore, they could not tell if a piece of gum, mouthwash, or lozenge contained more or less caffeine or even if any of the administration methods were caffeine-free. All participants completed the four acute protocols across four different and randomized weeks, following a previous protocol developed by our research group [23,24]. The same training protocol was used in all sessions (Figure 1). The random allocation sequence was computer generated (https://www.randomizer.org/). This study is reported following the extension of the Consolidated Standards of Reporting Trials (CONSORT) for randomized crossover trials [25].

### 2.1. Participants

Fourteen CrossFit^®^ males participated in this study (30.9 [5.62] years, 179 [1.33] cm, 78 [5.75] kg, 24.3 [1.33] kg·m^−2^). Eligible participants were aged 18–35 years with ≥6 months of continuous, structured CrossFit^®^ experience before study enrollment. All participants provided written informed consent after being informed of potential risks. The study protocol was approved by the University of Málaga Ethics Committee (code: 52-2025-H) and complied with the Declaration of Helsinki guidelines [26].

### 2.2. Intervention

#### 2.2.1. Exercise Program

Following the CAF administration, participants completed a standardized 15 min warm-up consisting of a 400 m medium-paced run followed by dynamic joint mobilization exercises targeting all major muscle groups (lower and upper body) [27]. The workout of the day (WOD) was the validated CrossFit^®^ ‘Cindy’ protocol [27], during which participants perform as many rounds as possible in 20 min of 5 pull-ups, 10 push-ups, and 15 air squats. Participants received technical instruction and practiced each movement comprising the subsequent work circuit. The ‘Cindy’ protocol was selected because its prolonged the duration, and high metabolic and perceptual demands align with the proposed ergogenic mechanisms of caffeine, particularly those related to central fatigue modulation and perceived exertion during sustained high-intensity exercise.

#### 2.2.2. Supplementation Protocol

For the CAF capsule condition, participants received a dose of 6 mg·kg^−1^ of anhydrous CAF (HSN Premium Raw Nutrition, Granada, Spain) 30 min prior to the WOD. In the CAF chewing gum (CCG) condition, participants chewed caffeinated gums (Blockhead Energy Gum, Leatherhead, UK), delivering 6 mg·kg^−1^ of CAF, from the beginning to the end of the WOD, following the manufacturer’s recommendations. The CAF mouth rinse (CMR) protocol involved rinsing with a solution containing 1.5 mg·kg^−1^ of anhydrous CAF (HSN Premium Raw Nutrition, Granada, Spain) dissolved in 25 mL of water with maltodextrin for 10 s. This rinse was performed at 5, 10, and 15 min during the WOD, totaling 6 mg·kg^−1^ of CAF. The placebo condition consisted of a sweetened commercial drink (Bolero^®^ Advanced Hydration, Edam, Netherlands), providing 6 mg·kg^−1^ of carbohydrates, consumed 30 min before the WOD. Participants were instructed to abstain from all other sources of CAF on the day of each testing session. To exclude a priori carryover effects, we used a 7-day washout period based on caffeine’s elimination half-life (~5 h) before each crossover (Figure 1). To ensure ecological validity, we replicated the real-world practice of CAF ingestion 30 min prior to training, mirroring typical athlete pre-workout routines. All assessments were conducted between 10 and 11 am, a time when a superior ergot effect has been demonstrated compared to nighttime administration. On the order hand, the different timing of caffeine administration across conditions reflects the distinct delivery strategies and proposed mechanisms of action of each method. However, this design does not allow for direct pharmacokinetic equivalence between conditions and should be considered when interpreting between-condition comparisons.

### 2.3. Outcomes

#### 2.3.1. Countermovement Jump (CMJ)

Lower-limb muscle power was assessed through countermovement jump height (cm) using the validated MyJump2^®^ application (My Jump 2, Madrid, Spain), version 2022 [28]. CMJ was included as a secondary outcome to assess neuromuscular function and potential caffeine-related effects on lower-limb power, rather than as a direct indicator of performance in the ‘Cindy’ WOD. Participants completed three maximal effort attempts per test, with 20 s rest intervals between jumps. The mean value of all three jumps (coefficient of variation = 4.55%) was used for statistical analysis, considering our group’s established methodology [29].

#### 2.3.2. Rate of Perceived Exertion (RPE)

To assess internal training load, participants rated their perceived exertion using a 10-point scale at two timepoints: immediately post-test and 15 min following completion of the Cindy protocol. We implemented our previously reported procedures [23,30] based on the original protocol [31].

#### 2.3.3. Total Number of Repetitions

The maximal number of repetitions was recorded for pull-ups, push-ups, and squats. Kipping technique was permitted for pull-ups. For push-ups, participants began in a plank position with elbows fully extended, required pectoral-to-floor contact at the bottom position, and had to return to full elbow extension without knee support. Squats demanded full knee extension at the top position and hip crease below knee level at the bottom. A CrossFit^®^ CF1-certified judge supervised all trials, counted valid repetitions, and disallowed attempts failing to meet movement standards. Prior to testing, participants received comprehensive movement demonstrations and technical instructions.

#### 2.3.4. Mean and Maximum Heart Rate

In a seated position, participants removed upper body clothing to facilitate proper placement of the Polar H10 heart rate sensor (Tampere, Finland). Following manufacturer guidelines, the electrode strap was moistened prior to application. To prevent behavioral influence, the sensor transmitted data to an external monitoring device rather than displaying real-time feedback to participants. All recordings were downloaded post-session for subsequent analysis.

### 2.4. Sample Size

The required sample size was calculated a priori using G*Power (v3.1.9.7) for a within-subjects repeated measures ANOVA with four conditions. Assuming a medium effect size (f = 0.25), α = 0.05, power = 0.80, a correlation of 0.5 between repeated measures, and no sphericity violations (ε = 1), the analysis indicated that a minimum of 12 participants was required. The crossover design allows each participant to serve as their own control, which supports adequate power for detecting between-condition differences despite a relatively small sample size. After the call to participate in this study, 24 subjects were potentially suitable for eligibility.

### 2.5. Statistical Analysis

All variables were expressed as means (standard deviation) and were analyzed using R v4.1 (R Core Team, 2021), with normality assessed via Shapiro–Wilk tests. Within-condition pre–post changes in CMJ were summarized as mean differences (Δ), visualized using estimation plots, and the smallest worthwhile change (SWC) was estimated as recommended for this variable when measured with a portable device; however, inferential conclusions were based on the results of the repeated measures analyses. Effect sizes for paired comparisons were calculated as unbiased Cohen’s *d* (d_unb_), interpreted as small (≤0.2), moderate (≈0.5), or large (≥0.8) [24]. For CMJ, a repeated measures analysis of variance (RM-ANOVA) was conducted with two within-subject factors: caffeine form (four levels: CMR, CCG, CC, and PG) and timepoint (pre, post, 15 min post). RPE was analyzed using the same factorial structure, with two timepoints (post and 15 min post). Total number of repetitions, HR_max_, and HR_mean_ were analyzed using one-way robust ANOVA. Greenhouse–Geisser corrections were applied when sphericity assumptions were violated, and Bonferroni-adjusted pairwise comparisons were used where appropriate. Finally, to validate the assumptions of the 4 × 4 crossover design, we assessed period, sequence, and carryover effects using a linear mixed model. Statistical significance was set at α = 0.05.

## 3. Results

### 3.1. Participant Flow

**Figure 2:**
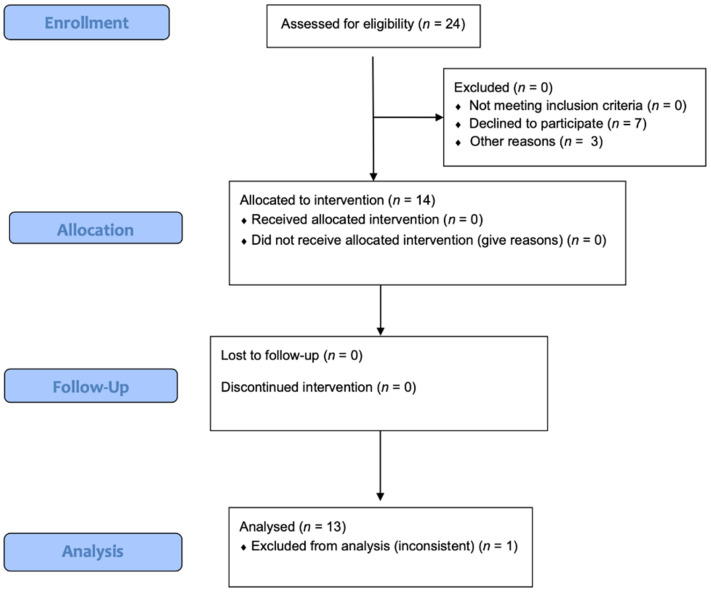
CONSORT diagram.

After the call to participate, 24 participants were potentially eligible. However, 10 individuals were excluded from this study due to declines to participate or other personal reasons. In addition, one participant was withdrawn from the analysis due to inconsistent data. Therefore, a total of 13 apparently healthy CrossFit^®^ participants (20–35 years of age) completed this crossover study. Figure 2 shows the CONSORT flow diagram.

### 3.2. Baseline Data

Participants’ baseline characteristics are presented in Table 1. The CAF supplementation was well tolerated among all participants (no side effects were reported).

### 3.3. Outcomes and Estimation

**Figure 3:**
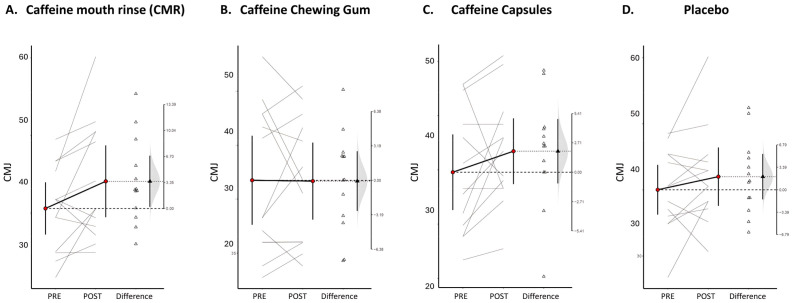
Estimation plots examining the within-participant comparisons for the CMJ. Paired data from different CAF forms and placebo conditions are shown as dots joined by gray lines. The differences between the pre- and post-intervention means are plotted on a floating difference axis, whose zero is aligned with the pre-intervention mean. The filled black triangle marks the difference on that axis, and the 95% CI on that difference is displayed. The differences are shown as open triangles on the difference axis.

The results of the within-participant comparison (analysis on paired data) for the CMJ are expressed as Δ (SD) [95% CI]; d_unb_ [95% CI]. Only participants of the CAF mouth rinse (CMR) intervention showed a potentially meaningful improvement, with the moderate effect size on lower-limb muscle power measured with the CMJ (+3.5 (5.46) [0.19, 6.80] cm; 0.51 [0.09, 1.02]), which exceeded the estimated SWC (1.2 cm) by approximately three-fold. No significant changes were observed in the CAF chewing gum (CCG: −0.07 (4.58) [−2.85, 2.69] cm; 0.01 [−0.42, 0.39]), CAF capsules (CC: +1.94 (4.92) [−1.03, 4.91] cm; 0.35 [−0.15, 0.91]), or placebo (+1.98 (5.69) [−1.46, 5.42] cm; 0.28 [−0.17, 0.79]) conditions. Figure 3 shows paired results between the initial and final measurements.

The repeated measures ANOVA revealed no significant main effects of the caffeine administration method on any measured outcomes. For the CMJ performance, no differences were observed across intake forms (F = 0.64, *p* = 0.700). Similarly, the RPE showed no interaction effects between the administration method and timepoint (F = 0.16, *p* = 0.922). Likewise, the total number of repetitions showed no significant time x group condition (F = 0.104, *p* = 0.957). Both the HR_max_ and HR_mean_ did not show relevant changes (HR_max_: F = 0.096, *p* = 0.961; HR_mean_: F = 0.279, *p* = 0.840). Within-condition pre–post changes in the CMJ are presented descriptively using mean differences and estimation plots to illustrate individual responses; however, all inferential conclusions regarding the effects of the caffeine form and time were based on the repeated measures ANOVA results. Accordingly, no significant main effects or interactions between caffeine forms and timepoints were observed for the CMJ or any other outcome. Complete results are presented in Table 2.

The results of the linear mixed model showed no significant carryover (Protocol × Period: *p* = 0.725), sequence (*p* = 0.844), or period effects (*p* = 0.605), validating our crossover design.

## 4. Discussion

To our knowledge this is the first study that investigated caffeine’s ergogenic effects when administered through different delivery methods (chewing gum [CCG], capsules [CCs], mouth rinse [CMR], and placebo) in CrossFit^®^ athletes. While we hypothesized that the CCG and CMR would outperform traditional capsules (CCs) and the placebo (PG), the results revealed a more nuanced pattern. Although no significant between-conditions differences emerged in the repeated measures ANOVA, the paired analysis showed a potentially meaningful improvement in the countermovement jump (CMJ) performance specifically with the CMR (+3.5 cm, d_unb_ = 0.51), suggesting a potential localized effect of buccal absorption on the explosive power output. This finding aligns partially with our hypothesis, as the rapid absorption through oral mucosa may have enhanced neuromuscular activation without systemic effects large enough to influence other performance metrics (repetitions, RPE, or HR parameters) [32,33].

Although a within-condition increase in the CMJ was descriptively observed following the caffeine mouth rinse (CMR) condition, this finding should not be interpreted as evidence of a superior or ergogenic effect. Importantly, the repeated measures ANOVA revealed no significant between-condition differences or interaction effects for the CMJ, indicating that no caffeine delivery form differed from the placebo or from each other. Therefore, emphasizing the within-condition CMJ change in isolation would be misleading. In the present manuscript, this result is reframed as exploratory and hypothesis-generating, serving primarily to inform future research rather than to support claims of efficacy or comparative advantage.

Although there is substantial research on CAF in various athletic populations [1,34,35,36], studies involving CrossFit^®^ participants remain limited. To date, only a few studies have examined the effects of CAF administration, mainly capsules, in CrossFit^®^ athletes. For instance, Fogaca et al. [20] evaluated the impact of anhydrous CAF on the strength, power, gymnastic movements, RPE, muscle soreness, and CMJ, among other parameters. Similar to our findings, no significant improvements were observed in performance, strength, power, or the RPE following CAF ingestion.

Likewise, Główka et al. [22] compared a CAF group and a placebo group in 26 moderately trained CrossFit^®^ practitioners. Their results showed no significant differences in the HR_max_, HR_mean_, RPE, or total repetitions—closely mirroring the outcomes of our study. In another investigation by Ziyaiyan and colleagues [19], CrossFit^®^ participants with more than two years of experience were assigned to one of three groups: CAF only, sodium bicarbonate, or a combination of both. These participants were evaluated using the Cindy protocol, as in our study. Performance measures, the RPE, muscle power, handgrip strength, and HR_max_ were assessed before and after the intervention. Again, no significant changes were observed with CAF alone; only the combination with sodium bicarbonate yielded improvements in the RPE compared to the control and placebo. Interestingly, the HR_max_ increased significantly in the CAF condition.

The study by Stein et al. [21] involved experienced CrossFit^®^ athletes performing as many repetitions as possible of squats, push-ups, and pull-ups over 20 min. Performance outcomes were not significantly different between the CAF and placebo conditions (468.6 ± 114.7 vs. 466.7 ± 94.3 repetitions, *p* = 0.861). More recently, Caetano and coworkers (2023) [36] administered 6 mg·kg−1 of CAF in capsule form to male CrossFit^®^ practitioners with at least two years of experience. The supplement was ingested 60 min before a squat test in a randomized, double-blind, placebo-controlled crossover trial. The CAF supplementation significantly increased the number of repetitions and total load lifted in a local muscular endurance test using a fixed percentage of the participant’s one repetition maximum (1RM). However, the authors did not find any benefit of CAF in increasing maximum strength, as measured by the 1RM squat test.

Taken together, these findings suggest that CAF does not consistently enhance performance or internal load indicators (such as RPE or HR_max_) in CrossFit^®^ contexts. In our study, although there was no significant effect on performance or physiological responses, a moderate effect size was observed for the CMJ performance only after the CRM intervention, suggesting a potential neuromuscular benefit that could hold practical relevance.

Similarly to Caetano et al. [37] but contrary to our findings, studies involving strength-trained individuals performing traditional resistance exercises have reported increased repetition counts following CAF ingestion [38]. One possible explanation for this discrepancy is the learning effect, as demonstrated by Stein and colleagues [21], where a significant increase in performance was seen between the first and second sessions regardless of treatment (452.4 ± 101 vs. 483.8 ± 106.5 repetitions, *p* = 0.001), with no effect of the treatment order (*p* = 0.438).

This learning phenomenon has been further supported by research such as de-Oliveira et al. [39]’s, which emphasized the importance of pacing strategies in WODs like AMRAP, EMOM, and FOR TIME. Such strategies can strongly influence performance and may reduce the observable impact of ergogenic aids like CAF. Finally, the mixed metabolic demands of CrossFit^®^ (compared to pure strength or endurance sports), suboptimal dosing timing (30 min vs. the conventional 60 min pre-exercise window), or potential ceiling effects in well-trained athletes may also help explain the limited efficacy of the CAF forms observed in our and other studies.

Although the crossover design enhances statistical power by reducing interindividual variability and the fact that our sample size (*n* = 13) was adequate for detecting the expected effect size, several limitations must be acknowledged: (i) the absence of pharmacokinetic measurements (e.g., caffeine plasma levels) to confirm absorption rates across different forms; (ii) no inclusion of female participants, limiting generalizability to sex-specific responses; (iii) the lack of strict control over participants’ total caffeine intake from other dietary sources beyond standardized pre-trial abstinence recommendations; and (iv) the lack of genotyping for the *CYP1A2* rs762551 polymorphism (163C > A), which influences caffeine metabolism—particularly given clinical evidence that ergogenic effects are more pronounced in A/A homozygotes compared to C allele carriers [40,41,42], with recent findings further supporting enhanced physical performance responsiveness in A allele carriers [43]. Future research might consider instrumental variable studies (e.g., Mendelian randomization) to estimate causal relationships [44]. On the other hand, while skipping pull-ups were allowed to reflect typical CrossFit^®^ practice, this movement may introduce greater technical variability compared to strict pull-ups. Such variability could have attenuated the detection of small ergogenic effects and should be considered when interpreting the results. Furthermore, due to the sensory characteristics of the chewing gum and mouthwash, the effectiveness of blinding may be considered a limitation.

An important methodological consideration of the present study is the assessment of multiple performance and physiological outcomes (CMJ, total repetitions, RPE, HR_mean_, and HR_max_) together with repeated within-condition comparisons. This analytical approach inherently increases the family-wise error rate and, consequently, the risk of Type I errors. Although correction procedures were applied within the primary analyses, isolated within-condition estimates should be interpreted with caution. Accordingly, any observed pre–post changes within individual caffeine conditions are considered exploratory and hypothesis-generating rather than confirmatory. The absence of significant main or interaction effects in the repeated measures ANOVA supports a conservative interpretation of the findings, and no claims of ergogenic efficacy are derived from isolated within-condition results.

## 5. Conclusions

Within the limitations of this study, acute caffeine ingestion at a dose of 6 mg·kg^−1^ body mass did not result in statistically significant improvements in internal load variables (RPE and heart rate) or total training volumes during a high-intensity functional training sessions in trained male CrossFit^®^ athletes. Although a small change in the countermovement jump performance was observed following the caffeine mouth rinse condition, this isolated finding was not accompanied by improvements in primary performances or physiological outcomes and should be interpreted with caution. Given the small sample size, the male-only cohort, and the lack of strict control of caffeine habituation, these findings should be considered hypothesis-generating rather than conclusive. Future studies with larger and more diverse samples are warranted.

## Figures and Tables

**Table 1:** Participants’ baseline characteristics.

Variable	$\bar{X}$	SD	95% CI (LL, UL)
Age (years)	30.9	5.62	27.7, 34.2
Stature (cm)	179.0	4.22	176.5, 181.4
Body mass (kg)	78.0	5.75	74.7, 81.3
Body mass index (kg·m^−2^)	24.3	1.33	23.5, 25.1

Note: Data are expressed as mean (standard deviation). CI: confidence interval; LL: lower limit; and UL: upper limit.

**Table 2:** Main effects on performance and physiological responses across different caffeine forms.

	CMR	CCG	CC	PG	Main Effects		ES
	$\bar{X}$ (SD)	$\bar{X}$ (SD)	$\bar{X}$ (SD)	$\bar{X}$ (SD)	F	*p*	
CMJ_Pre_	39.6 (6.51)	40.9 (7.31)	38.8 (6.57)	39.3 (6.59)	1.11	0.36	
CMJ_Post_	42.7 (8.99)	39.7 (9.26)	40.3 (7.29)	40.5 (9.08)			η^2^_p_ = 0.038
CMJ_Post_15m_	39.9 (5.32)	39.3 (7.82)	38.7 (6.72)	38.7 (5.81)			
Repetitions	555 (152)	556 (147)	550 (157)	560 (130)	0.01	0.97	ξ = 0.111
RPE_Post_	8.21 (1.12)	8.43 (1.09)	8.43 (0.852)	8.29 (1.27)	0.10	0.96	η^2^_p_ = 0.01
RPE_Post_15m_	7.43 (1.45)	7.71 (1.33)	7.71 (1.14)	7.43 (1.22)			
HR_mean_	152 (11.1)	150 (15.6)	153 (14.0)	152 (138)	0.20	0.91	ξ = 0.147
HR_max_	186 (9.09)	187 (8.11)	185 (11.9)	187 (7.78)	0.93	0.93	ξ = 0.098

Note: η^2^_p_: partial eta squared (effect size of the repeated measures ANOVA); ξ: Cohen’s ξ (effect size of the robust ANOVA with trimmed means); CC: caffeine capsule; CCG: caffeine chewing gum; CMJ: countermovement jump; CMR: caffeine mouth rinse; ES, effect size; HR: heart rate; PG: placebo group; and RPE: rate of perceived exertion.

## Data Availability

The data presented in this study are available on request from the corresponding authors (benitez@uma.es).

## Abbreviations

The following abbreviations are used in this manuscript:

CAF	Caffeine
CC	Caffeine capsule
CCG	Caffeine chewing gum
CMR	Caffeine mouth rinse
PG	Placebo group
CMJ	Countermovement jump
RPE	Rate of perceived exertion
HR	Heart rate
HR_max_	Maximum heart rate
HR_mean_	Mean heart rate
WOD	Workout of the day
CI	Confidence interval
SD	Standard deviation
RM	Repetition maximum
ANOVA	Analysis of variance
CONSORT	Consolidated Standards of Reporting Trials
